# Diffusion weighted imaging in high-grade gliomas: A histogram-based analysis of apparent diffusion coefficient profile

**DOI:** 10.1371/journal.pone.0249878

**Published:** 2021-04-15

**Authors:** Georg Gihr, Diana Horvath-Rizea, Elena Hekeler, Oliver Ganslandt, Hans Henkes, Karl-Titus Hoffmann, Cordula Scherlach, Stefan Schob

**Affiliations:** 1 Clinic for Neuroradiology, Katharinenhospital Stuttgart, Stuttgart, Germany; 2 Department for Pathology, Katharinenhospital Stuttgart, Stuttgart, Germany; 3 Clinic for Neurosurgery, Katharinenhospital Stuttgart, Stuttgart, Germany; 4 Department for Neuroradiology, University Hospital Leipzig, Leipzig, Germany; 5 Department for Radiology, University Hospital Halle (Saale), Halle (Saale), Germany; Northwestern University Feinberg School of Medicine, UNITED STATES

## Abstract

**Purpose:**

Glioblastoma and anaplastic astrocytoma represent the most commonly encountered high-grade-glioma (HGG) in adults. Although both neoplasms are very distinct entities in context of epidemiology, clinical course and prognosis, their appearance in conventional magnetic resonance imaging (MRI) is very similar. In search for additional information aiding the distinction of potentially confusable neoplasms, histogram analysis of apparent diffusion coefficient (ADC) maps recently proved to be auxiliary in a number of entities. Therefore, our present exploratory retrospective study investigated whether ADC histogram profile parameters differ significantly between anaplastic astrocytoma and glioblastoma, reflect the proliferation index Ki-67, or are associated with the prognostic relevant MGMT (methylguanine-DNA methyl-transferase) promotor methylation status.

**Methods:**

Pre-surgical ADC volumes of 56 HGG patients were analyzed by histogram-profiling. Association between extracted histogram parameters and neuropathology including WHO-grade, Ki-67 expression and MGMT promotor methylation status was investigated due to comparative and correlative statistics.

**Results:**

Grade IV gliomas were more heterogeneous than grade III tumors. More specifically, ADCmin and the lowest percentile ADCp10 were significantly lower, whereas ADCmax, ADC standard deviation and Skewness were significantly higher in the glioblastoma group. ADCmin, ADCmax, ADC standard deviation, Kurtosis and Entropy of ADC histogram were significantly correlated with Ki-67 expression. No significant difference could be revealed by comparison of ADC histogram parameters between MGMT promotor methylated and unmethylated HGG.

**Conclusions:**

ADC histogram parameters differ significantly between glioblastoma and anaplastic astrocytoma and show distinct associations with the proliferative activity in both HGG. Our results suggest ADC histogram profiling as promising biomarker for differentiation of both, however, further studies with prospective multicenter design are wanted to confirm and further elaborate this hypothesis.

## Introduction

Gliomas belong to the group of primary central nervous system (CNS) neoplasias and develop from glial cells of the CNS. According to epidemiologic studies, they account for almost one third of all diagnosed brain tumors in adults [1]. The World Health Organization (WHO) classification system categorizes gliomas from the lowest grade WHO I to the highest grade WHO IV [2], mainly based on histopathological tissue properties such as cell proliferation, tissue necrosis and cell pleomorphism. Grade WHO I and II represent tumor entities with a rather benign tumor biology and are classified as low-grade gliomas (LGG). Whereas entities of category WHO III and IV are considered as malign lesions, exhibiting aggressive tumor biology and therefore classified as high-grade gliomas (HGG).

The two most common HGG in adults are the glioblastoma (WHO IV) with about 14.9% and the anaplastic astrocytoma with about 1.7% of all newly diagnosed intracranial masses [1]. Glioblastoma, or astrocytoma WHO grade IV—the most fatal brain tumor in humans—remains associated with a median overall survival of 15 months [3] despite significant advances in understanding the underlying genetic, epigenetic and downstream alterations in the last decade [4]. In contrast to glioblastoma, anaplastic astrocytoma (WHO III) exhibits a distinctly better prognosis with a median overall survival of 2 to 10 years, depending on associated genetic alterations like isocitrate dehydrogenase (IDH) mutation [2, 5, 6].

Magnetic resonance imaging (MRI), with its unparalleled soft tissue contrast, is the most important imaging modality for all intracranial masses. In case of HGG, routinely used morphological MRI sequences like T2-weighted and contrast enhanced T1-weighted sequences are pivotal for the precise definition of tumor localization, evaluation of its mass effect, necrosis and peritumoral edema—information being essential to plan the surgical therapy. However, qualitative morphological data remain rather unreliable concerning the differentiation between grade III and grade IV astrocytoma, not least because of the regularly similar-looking appearance on such MRI images. Therefore, advanced MRI techniques, including MR-spectroscopy, perfusion-weighted imaging, and diffusion-weighted imaging are complementarily used in most of the standard MRI protocols in order to improve the diagnostic accuracy.

Especially diffusion-weighted imaging (DWI) is a well-established and nonetheless still evolving technique in the MRI domain. DWI is able to estimate the random diffusional motion of water molecules [7] in biological tissues and enables calculation and mapping of the apparent diffusion coefficient (ADC) of the respective target volume in vivo. Since ADC tumor profiles reflect the corresponding microscopic tissue architecture [8], DWI has evolved to an important MRI modality especially in cancer imaging [9]. In case of gliomas, DWI and ADC-mapping have shown promising results in terms of estimating the tumor growth potential [10] and clinical prognosis of glioma patients [11] as well as for distinguishing between HGG and brain abscess [12]. However, most of the underlying DWI studies investigated only the first order ADC histogram parameters, including mean, median, minimum, maximum as well as percentiles and disregarded the so called second order characteristics obtained by a complete histogram analysis (HA), namely skewness, kurtosis and entropy. These second order histogram characteristics provide additional information about value distribution and thus basically better reflect tumor heterogeneity [13]. Several whole tumor HA studies of ADC profiles, published over the last years, indicated the utility and diagnostic potential of this approach, especially in terms of tumor grading and estimating the proliferative potential of the tumor tissue and therefore also the clinical prognosis [14–20]. Using both, the aforementioned first and second order characteristics, even HA of simple MRI signal intensities, obtained from conventional sequences, enabled to draw conclusions about the underlying tumor histopathology [21–24]. In summary, it can be stated that whole tumor HA of data obtained by MRI is a promising, capable and easily available radiomic approach, providing additional non-morphological information in contrast to conventional MRI images.

Therefore, the aim of this exploratory retrospective study was to evaluate whether whole tumor HA of ADC profiles has the ability to i) differentiate WHO grade III anaplastic astrocytoma from WHO grade IV glioblastoma, to ii) estimate the proliferative potential of the neoplasms represented by the Ki-67 proliferation index and to iii) predict the prognostic relevant MGMT (methylguanine-DNA methyl-transferase) promotor methylation status.

## Materials and methods

### Ethics statement

The study was approved by the ethics committee of the medical council of Baden-Württemberg (Ethik-Kommission Landesärztekammer Baden-Württemberg, F-2017-047). The patient data used in our study were extracted from the hospital`s electronic medical records system (Katharinenhospital Stuttgart, Stuttgart, Germany) in the period between 01/2012 and 02/2017 after full anonymization.

### Patients collective

Our institutional radiological information system (RIS) was searched for matching patients using the search criteria glioma and primary brain tumor. A total of 72 patients were identified in the period between 01/2012 and 02/2017. All patients underwent either a diagnostic biopsy or tumor resection in our hospital with subsequent neuropathological workup of the tissue samples. Histological diagnosis, MGMT promotor methylation status and the proliferation index Ki-67 were taken from the hospital patient database. Only patients with presurgical MRI examination and sufficient DWI datasets were included in the study. MRI scans were checked for hemorrhage, significant calcifications or artificial data due to other causes and excluded if necessary, to avoid artifact-related distortion of ADC values. Finally, 56 patients (11 anaplastic astrocytomas and 45 glioblastomas, 22 females, 34 males; with a mean age of 62 years) were included in our retrospective analysis.

### MRI specifics

All patients received an MRI scan of the brain using a 1.5 T device (MAGNETOM Aera and MAGNETOM Symphony Tx/Rx CP head coil, Siemens, Erlangen, Germany). The following imaging protocol was used:

1. axial T1 weighted (T1w) spin echo (SE) sequences (TR/TE: 453/17, flip angle: 90°, slice thickness: 5mm, acquisition matrix: 320x179, field of view: 230x187 mm) prior and post intravenous application of contrast medium (Gadobutrol, Gadovist®, Bayer Schering Pharma, Leverkusen, Germany)
2. axial DWI (readout-segmented, multi-shot EPI sequence; TR/TE: 5500/103, flip angle 90°, slice thickness: 5mm, acquisition matrix: 152x144, field of view: 230 x 230 mm) with b values of 0 and 1000 s/mm^2^. ADC maps were automatically calculated by the pre-installed software package of the device manufacturer.

All MRI images and ADC maps were available in digital form and subsequently analyzed by two experienced radiologists (DHR, SS) on a PACS workstation (Impax EE R20 XII) without knowledge of the histopathological diagnosis.

### Histogram profiling of ADC maps

After exporting the ADC maps and T1 weighted images from our institutional archive in a DICOM format *via* the aforementioned AGFA PACS, whole lesion histogram profiling was carried out by using a custom-made DICOM image analysis tool (programmed by N.G. using Matlab, The Mathworks, Natick, MA) as follows: Initially, T1weighted images were loaded into a graphical user interface (GUI) to mark the tumor suspected lesion of each patient in all respective MRI sections. Then, all regions of interest (ROIs) were automatically co-registered with the corresponding ADC maps, followed by whole lesion histogram profiling with calculation of ADCmean, ADCmin, ADCmax, ADCp10, ADCp25, ADCp75, ADCp90, ADCmodus, ADCmedian, ADC standard deviation (SD), Skewness, Kurtosis and Entropy. All primary histogram data on each patient level as well as the corresponding histopathological information are provided in S1 Table. The complete MRI-dataset can be obtained via the public repository Zenodo (doi: 10.5281/zenodo.4605319).

### Neuropathology

For histological diagnostics, immunohistochemistry and PCR sequencing, the tumor specimens were formalin-fixed and paraffin-embedded. In a first step, the embedded samples were sectioned at 3μm, followed by hematoxylin and eosin (H&E) staining as well as immunohistochemistry using specific antibodies against Ki-67 (product number: M7240; dilution 1: 800; Dako Denmark A/S, Glostrup, Denmark). All sectioned tissue samples were analyzed histologically for presence of viable tumor infiltration, absence of significant necrosis and hemorrhage prior to further processing. Digitalization of the histopathological images was performed with a Leica microscope, carrying a DFC290 HD digital camera and LAS V4.4 software (Leica Microsystems, Wetzlar, Germany). Tumor proliferation index was estimated by dividing the number of specifically stained (Ki-67 positive) cell nuclei by all nuclei. The area showing the highest number of positive cell nuclei was selected in each case.

In a second step, the paraffin-embedded tumor samples were dissected into 10 μm slices for DNA isolation using the Maxwell® RSC FFPE Plus DNA Kit AS1720 (Promega, USA) with a Maxwell® RSC Instrument (Promega, USA). Conversion of unmethylated cytosine residues to uracil was performed by bisulfite treatment using the EpiTect® Bisulfite Kit (QIAGEN, Germany), each step according to the manufacturer’s procedures. Subsequently, the bisulfite-converted DNA was amplified via PCR reaction and the methylation status of the MGMT gene was determined by pyrosequencing according to the manufacturer’s protocol using the Therascreen MGMT Pyro® Kit (QIAGEN, Germany), testing 4 CpG islands (chromosome 10, Exon 1, range 131265519–131265537, CGACGCCCGCAGGTCCTCG). Methylation percentage of 10% and higher was considered as methylation positive.

### Statistical analysis

Statistical analysis including graphics creation was performed using GraphPad Prism 8 (GraphPad Software, San Diego, CA, USA).

After investigating the DWI data and histopathological information by descriptive statistics, the data was tested for Gaussian distribution by using the Shapiro-Wilk-Test. To compare normally distributed DWI histogram profiling parameters between grade III and grade IV astrocytoma as well as between MGMT promotor methylated and unmethylated gliomas, T-Test was performed. Comparison of parameters showing a non-Gaussian distribution between grade III and grade IV and between MGMT promotor methylated and unmethylated astrocytoma was performed by using the Mann-Whitney-U Test. For Correlation analysis the Pearson Correlation Coefficient was used in case of normally distributed parameters and the Spearman-Rho Rank-Order Correlation Calculation in case of non-Gaussian distribution. Generally, p-values < 0,05 were taken to indicate statistical significance in all instances.

Finally, receiver operating characteristics (ROC) curve analysis was performed and the respective area under the curve (AUC) was calculated to assess the accuracy of ADC volume histogram profiling. Youden’s Index was calculated for those ADC parameters with the best test accuracy to estimate possible cut-off values.

## Results

**Fig 1:**
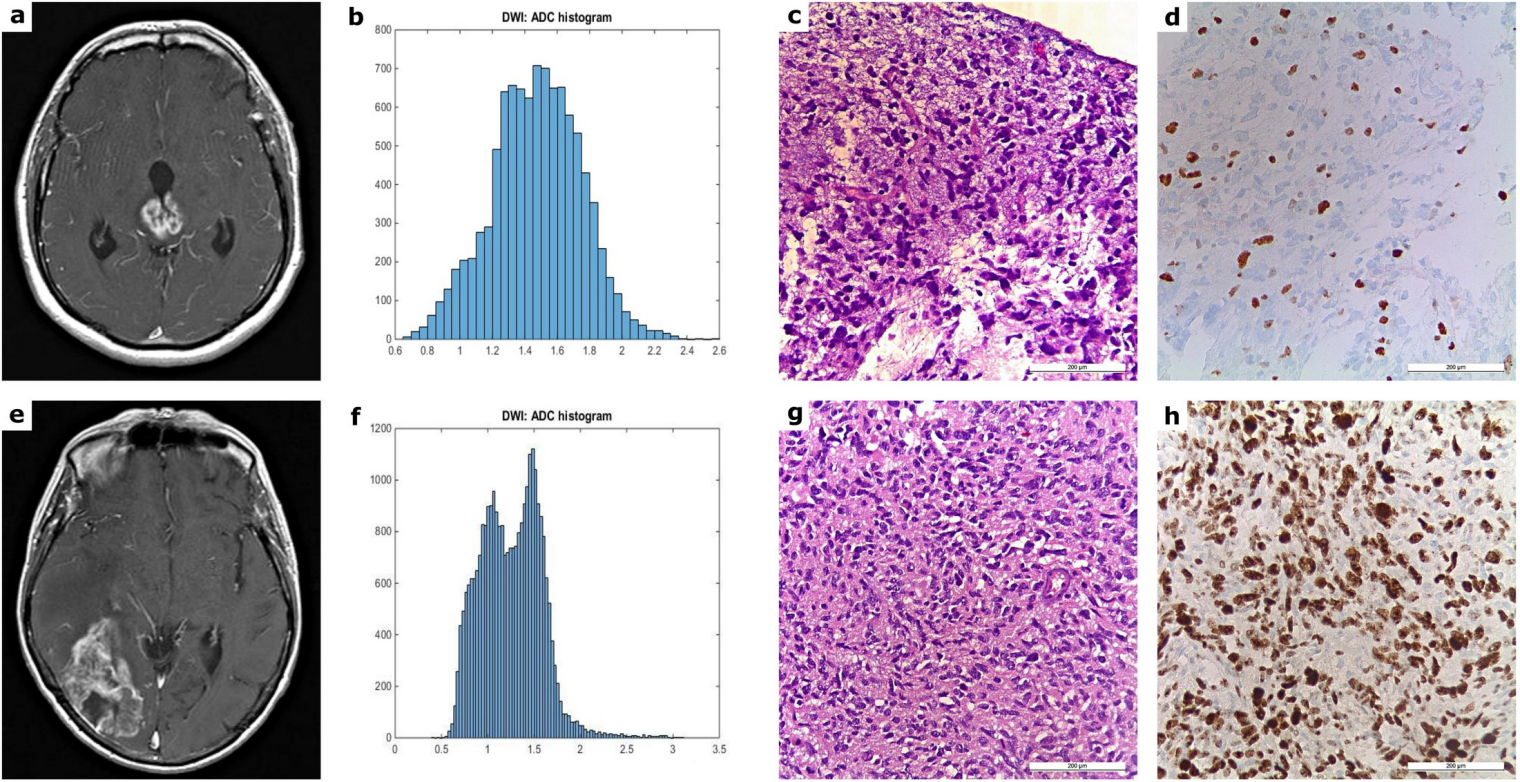
MRI, ADC histogram and histopathological findings in patients with HGG. Fig 1 shows typical MRI images, the inherent tumor volume ADC histogram as well as H&E staining and Ki-67 immunohistochemistry of a grade III (a-d) and a grade IV glioma (e-h). The first image of the upper case displays a T1 weighted turbo-spin-echo (TSE) sequence (after intravenous application of a gadolinium-based contrast medium) of a grade III astrocytoma, involving the right and left thalamus as well as the aqueduct with consecutive hydrocephalus (a). The first image of the lower case illustrates a contrast enhanced T1 weighted TSE sequence of a grade IV glioblastoma of the right occipital and the adjacent temporal lobe with marked perifocal edema and mass effect (e). Each MRI example is followed by the corresponding ADC histogram (b, f; x-axis: ADC values in incremental order, y-axis: number of voxels), the H&E staining and the Ki-67 immunohistochemistry on the right side (c-d, g-h). A proliferation index of 12% was calculated for the anaplastic astrocytoma and a proliferation index of 80% for the glioblastoma.

Brain MRI scans from patients with HGG, the corresponding tumor volume ADC histograms as well as the histopathological images (HE and Ki-67) are displayed in Fig 1.

Table 1 summarizes the results of the descriptive statistics of DWI histogram parameters concerning all investigated gliomas. Values for ADCmean, ADCp10, ADCp25, Skewness and Entropy showed a Gaussian distribution (all p < 0.05), whereas non-Gaussian distribution was revealed for ADCmin, ADCmax, ADCp75, ADCp90, ADCmedian, ADCmodus, ADC SD, Kurtosis, Skewness and Ki-67.

**Table 1:** DWI histogram profiling parameters of all investigated high-grade gliomas.

Parameters	Mean ± SD	Minimum	Maximum
**ADC**_**mean**_**,** × 10^−5^ mm^2^s^-1^	137.57 ± 31.60	66.56	218.48
**ADC**_**min**_**,** × 10^−5^ mm^2^s^-1^	39.48 ± 28.70	0.10	101.30
**ADC**_**max**_**,** × 10^−5^ mm^2^s^-1^	286.62 ± 59.31	107.30	397.80
**P10 ADC,** × 10^−5^ mm^2^s^-1^	95.59 ± 20.70	44.50	160.70
**P25 ADC,** × 10^−5^ mm^2^s^-1^	109.61 ± 24.25	49.80	179.90
**P75 ADC,** × 10^−5^ mm^2^s^-1^	162.08 ± 46.16	75.30	274.00
**P90 ADC,** × 10^−5^ mm^2^s^-1^	186.73 ± 49.76	82.79	283.70
**Median ADC,** × 10^−5^ mm^2^s^-1^	132.42 ± 34.90	59.85	255.80
**Mode ADC,** × 10^−5^ mm^2^s^-1^	130.43 ± 54.11	46.30	277.00
**SD ADC,** × 10^−5^ mm^2^s^-1^	37.07 ± 16.05	8.77	77.46
**Kurtosis**	4.94 ± 3.99	1.35	23.34
**Skewness**	0.72 ± 0.95	-2.07	3.80
**Entropy**	4.73 ± 0.51	3.25	5.55

Table 1 shows the results of the descriptive ADC histogram analysis of all investigated gliomas.

**Fig 2:**
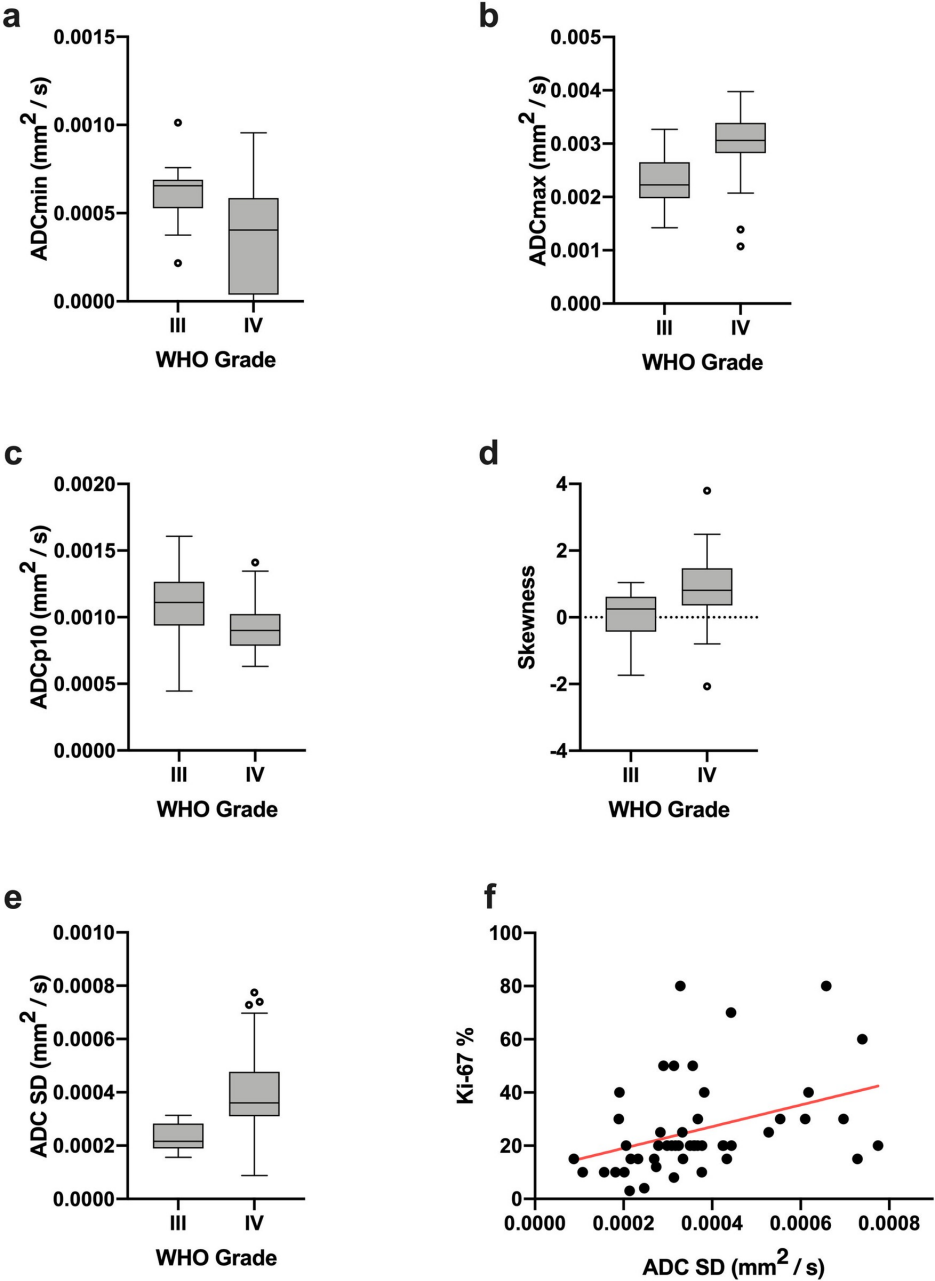
Significant different ADC histogram parameters between grade III and IV gliomas, correlation between ADC SD and Ki-67. Fig 2 displays boxplots of ADC histogram parameters that differed significantly between grade III and grade IV gliomas (a-e). The strongest correlation (between ADC SD of the whole tumor ADC histograms and the proliferation index Ki-67; r = 0.4608, p = 0.0008) is illustrated in image (f).

In brief, comparative analysis detected significant differences between grade III and grade IV astrocytomas for ADCmin, ADCmax, ADCp10, ADC SD and Skewness (all p < 0.05). Mean values of ADCmin and the lowest percentile ADCp10 were significantly lower in the WHO grade IV group, whereas ADCmax, ADC SD and Skewness were significantly lower in the WHO grade III group. As expected, WHO grade IV astrocytomas showed significant higher values for the Ki-67 proliferation index compared to their WHO grade III counterparts. On the contrary, no significant difference could be revealed by comparison of ADC histogram parameters between MGMT promotor methylated and unmethylated HGG. The results of the complete comparative statistical analysis are provided in Tables 2 and 3. Boxplots of ADC histogram profile parameters that achieved statistically significant differences between WHO grade III and IV astrocytomas are illustrated in Fig 2A–2E.

**Table 2:** Comparison of DWI histogram profiles and Ki-67 index between grade III and grade IV glioma.

Parameters	WHO Grade 3 Mean (SD) / 95%-CI		WHO Grade 4 Mean (SD) / 95%-CI		p-values
**ADC**_**mean**_**,** × 10^−5^ mm^2^s^-1^	136.90 (29.88)	116.84–156.99	137.70 (32.68)	127.92–147.55	0.9398
**ADC**_**min**_**,** × 10^−5^ mm^2^s^-1^	61.29 (6.17)	47.55–75.02	34.14 (4.23)	25.62–42.67	**0.0040**
**ADC**_**max**_**,** × 10^−5^ mm^2^s^-1^	231.90 (49.08)	198.89–264.84	300.00 (54.75)	283.56–316.46	**0.0001**
**P10 ADC,** × 10^−5^ mm^2^s^-1^	108.60 (8.87)	88.87–128.40	92.40 (2.56)	87.24–97.56	**0.0194**
**P25 ADC,** × 10^−5^ mm^2^s^-1^	121.10 (32.91)	99.00–143.21	106.80 (21.48)	100.34–113.25	0.0820
**P75 ADC,** × 10^−5^ mm^2^s^-1^	153.00 (30.24)	132.65–173.28	164.30 (49.78)	149.36–179.27	0.9639
**P90 ADC,** × 10^−5^ mm^2^s^-1^	166.70 (26.29)	149.02–184.35	191.60 (53.57)	175.53–207.72	0.2507
**Median ADC,** × 10^−5^ mm^2^s^-1^	135.70 (33.96)	112.85–158.48	131.60 (35.84)	120.86–142.39	0.2772
**Mode ADC,** × 10^−5^ mm^2^s^-1^	136.50 (40.47)	109.29–163.66	129.00 (57.81)	111.58–146.32	0.1032
**SD ADC,** × 10^−5^ mm^2^s^-1^	23.55 (5.42)	19.91–27.20	40.38 (16.25)	35.50–45.26	**<0.0001**
**Kurtosis**	3.97 (1.67)	2.84–5.09	5.18 (4.40)	3.86–6.50	0.7759
**Skewness**	0.07 (0.79)	-0.46–0.60	0.88 (0.94)	0.60–1.17	**0.0108**
**Entropy**	4.77 (0.39)	4.51–5.03	4.72 (0.54)	4.55–4.88	0.7561
**Ki-67**	15.20 (14.33)	4.95–25.45	28.38 (17.77)	22.71–34.06	**0.0012**

Table 2 compares DWI histogram profiling values of WHO grade III and WHO grade IV gliomas. P-values of statistically different comparisons are given in bold writing.

**Table 3:** Comparison of DWI histogram profiles between high-grade gliomas with and without MGMT promotor methylation.

Parameters	MGMT promotor methylation positive Mean (SD) / 95%-CI		MGMT promotor methylation negative Mean (SD) / 95%-CI		p-values
**ADC**_**mean**_**,** × 10^−5^ mm^2^s^-1^	133.50 (31.62)	119.19–147.88	139.30 (31.93)	127.16–151.45	0.5258
**ADC**_**min**_**,** × 10^−5^ mm^2^s^-1^	42.16 (29.67)	28.66–55.67	35.57 (28.81)	24.61–46.53	0.4208
**ADC**_**max**_**,** × 10^−5^ mm^2^s^-1^	277.70 (59.54)	250.60–304.80	291.90 (61.92)	268.33–315.43	0.3074
**P10 ADC,** × 10^−5^ mm^2^s^-1^	94.83 (22.31)	84.67–104.98	95.08 (20.66)	87.22–102.94	0.7442
**P25 ADC,** × 10^−5^ mm^2^s^-1^	106.90 (25.01)	95.51–118.27	110.70 (25.06)	101.20–120.26	0.5951
**P75 ADC,** × 10^−5^ mm^2^s^-1^	157.40 (46.59)	136.24–178.66	164.20 (46.06)	146.70–181.73	0.9650
**P90 ADC,** × 10^−5^ mm^2^s^-1^	183.70 (52.29)	159.88–207.48	189.00 (50.06)	169.95–208.03	0.9070
**Median ADC,** × 10^−5^ mm^2^s^-1^	125.40 (29.87)	111.78–138.98	135.20 (36.59)	121.29–149.13	0.7739
**Mode ADC,** × 10^−5^ mm^2^s^-1^	119.70 (46.31)	98.64–140.80	134.70 (58.25)	112.55–156.87	0.5424
**SD ADC,** × 10^−5^ mm^2^s^-1^	35.99 (15.59)	28.89–43.08	37.93 (16.87)	31.51–44.34	0.6808
**Kurtosis**	4.36 (2.41)	3.26–5.45	5.01 (3.80)	3.56–6.45	0.9534
**Skewness**	0.87 (0.68)	0.56–1.18	0.58 (1.02)	0.19–0.97	0.2737
**Entropy**	4.79 (0.52)	4.38–4.92	4.84 (0.45)	4.67–5.01	0.9789

Table 3 compares DWI histogram profiling values of MGMT promotor methylation positive and negative gliomas.

Statistical correlation analysis, using the Pearson’s Correlation Coefficient in case of normal distributed data and the Spearman-Rho Rank-Order Correlation in case of non-Gaussian distribution, identified significant associations (p < 0.05) between the proliferation index Ki-67 and ADCmin, ADCmax, ADC SD, Kurtosis and Entropy. The results of the correlative analysis are represented in Table 4. ADC SD and Ki-67 exhibited the strongest correlation (r = 0.4608, p = 0.0008), the scatter plot of this association is diagrammed in Fig 2F.

**Table 4:** Correlations between DWI histogram profile parameters and Ki-67 in all investigated gliomas.

DWI Histogram Profile Parameters	Ki-67
**ADC**_**mean**_**,** × 10^−5^ mm^2^s^-1^	r = 0.01371
	p = 0.9247
**ADC**_**min**_**,** × 10^−5^ mm^2^s^-1^	**r = -0.3037**
	**p = 0.0320**
**ADC**_**max**_**,** × 10^−5^ mm^2^s^-1^	**r = 0.4083**
	**p = 0.0032**
**ADCp10,** × 10^−5^ mm^2^s^-1^	r = -0.1808
	p = 0.2090
**ADCp25,** × 10^−5^ mm^2^s^-1^	r = -0.1459
	p = 0.3119
**ADCp75,** × 10^−5^ mm^2^s^-1^	r = 0.06448
	p = 0.6564
**ADCp90,** × 10^−5^ mm^2^s^-1^	r = 0.2135
	p = 0.1366
**ADCMedian,** × 10^−5^ mm^2^s^-1^	r = -0.06758
	p = 0.6410
**ADCModus,** × 10^−5^ mm^2^s^-1^	r = -0.02073
	p = 0.8864
**SD ADC,** × 10^−5^ mm^2^s^-1^	**r = 0.4608**
	**p = 0.0008**
**Kurtosis**	**r = -0.2846**
	**p = 0.0452**
**Skewness**	r = 0.1705
	p = 0.2366
**Entropy**	**r = 0.2866**
	**p = 0.0436**

Table 4 summarizes the correlative analysis of DWI histogram profiling and Ki-67. Significant results are given in bold writing.

**Fig 3:**
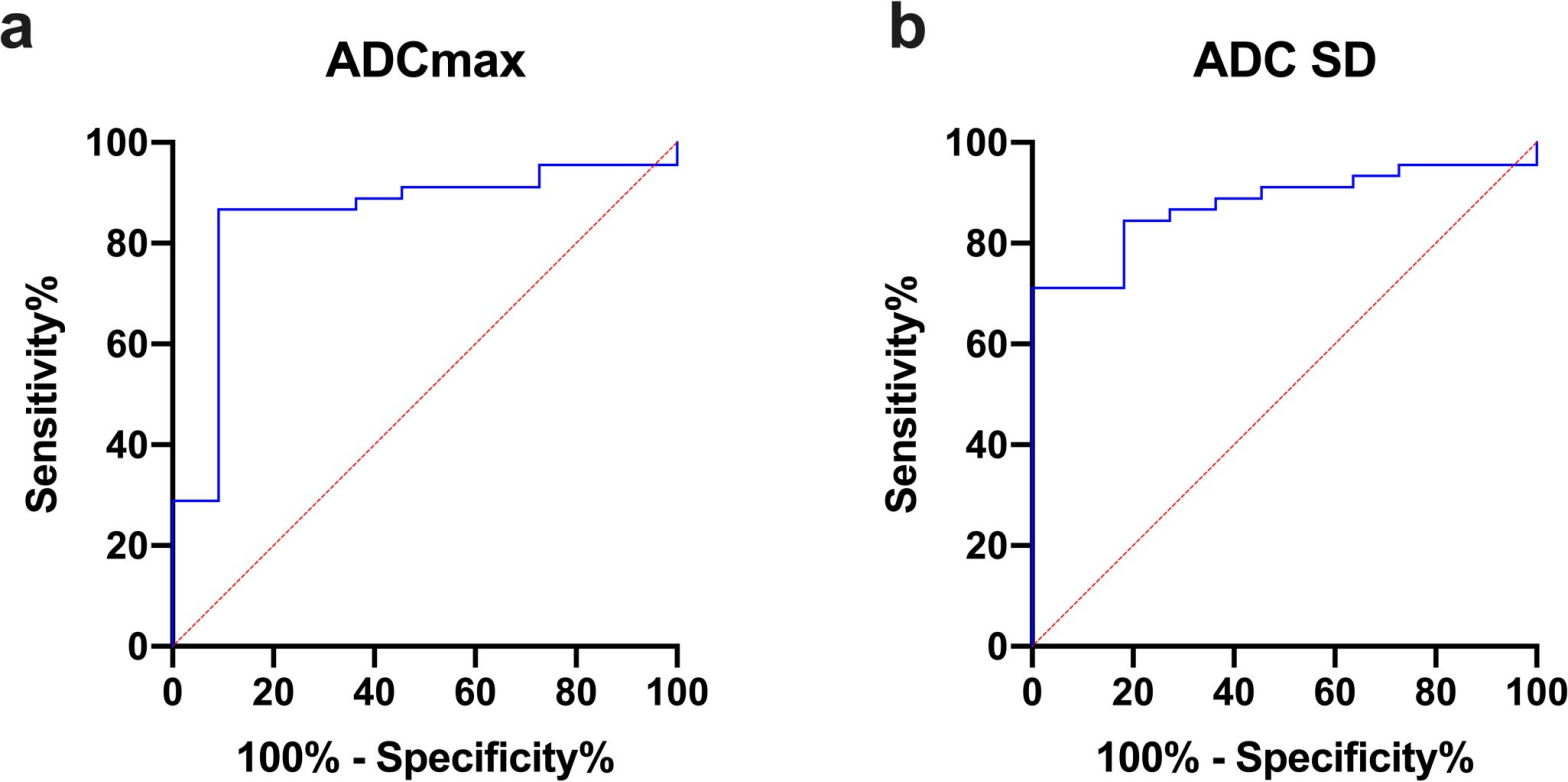
ROC curves. Fig 3 shows the receiver operating characteristics (ROC) curve of ADCmax (a) and ADC SD (b), the parameters with the highest AUC values and therefore the best accuracy in terms of discrimination between grade III and grade IV gliomas.

Moreover, to evaluate the potential test accuracy in terms of differentiating grade III from grade IV glioma AUC values were calculated for each of the histogram parameters that reached significance in the comparative statistical analysis. The following values were obtained (CI: confidence interval): ADCmin (AUC = 0.7747, [CI: 0.6373–0.9122], p = 0.005), ADCmax (AUC = 0.8525, [CI: 0.7231–0.9819], p = 0.0003), ADCp10 (AUC = 0.7374, [CI: 0.5524–0.9224], p = 0.0154), ADC SD (AUC = 0.8768, [CI: 0.7858–0.9677], p = 0.0001) and Skewness (AUC = 0.7596, [CI: 0.6159–0.9033], p = 0.0080). The ROC of the two parameters with the best accuracy, ADCmax and ADC SD, are shown in Fig 3. The potential cut-off value was finally calculated for ADC SD via Youden’s Index: ADC SD values of 0.0003156 and greater indicate grade IV astrocytoma (sensitivity: 0.71, specificity: 1.00).

## Discussion

Anaplastic astrocytoma (WHO grade III) and glioblastoma (WHO grade IV) exhibit a highly heterogeneous tissue microarchitecture with areas of very distinct mitotic activity and cellularity [25].

It has exemplarily been reported that the so called peritumoral zone, which is morphologically characterized by vasogenic edema, but does not yet reveal blood brain barrier disruption, contains especially aggressive and highly proliferating tumor cell islets, being causative for local recurrence in most cases [25]. Unfortunately, those areas, representing important surgical targets, remain unremarkable in conventional imaging. As a consequence, the identification of those neoplastic spots with increased proliferation, which are predominantly located inside the contrast enhancing tumor volume, but also occur in the surrounding peritumoral zone, is pivotal for accurate biopsy results and, even more importantly, optimized surgery. Since contrast enhanced T1w images, T2w- and FLAIRw images do not reliably identify those biologically determinative tumor components, DWI represents the technique of choice in this particular context.

The ADC map of a tumor reflects its proliferative activity and cellular density, with decreased ADC values revealing increased cellularity and higher tumor grades in most entities [19, 20, 26–28]. Nevertheless, a two-dimensional ROI on the ADC cannot sufficiently transfer the whole spectrum of information related to the complex micro-architecture of malignantly transformed tissue, but rather fabricates a filtered and compressed approximation, which limits the potential of the ADC as oncologic imaging biomarker [29]. Therefore, application of histogram analysis of ADC maps or a comparatively precise investigational tool is paramount to achieve reliable quantitative features that facilitate optimal evaluation of a tumor´s microarchitecture including areas of increased mitotic activity.

Our present study revealed that the lowest percentile of the ADC continuum, ADCp10, as well as the maximum and minimum values, ADCmax and ADCmin, significantly differed between grade III and IV gliomas. More specifically, grade IV gliomas in our cohort had lower ADCmin- and higher ADCmax-values, reflecting broader tumor heterogeneity compared to grade III lesions. This hypothesis is corroborated by the fact that grade IV gliomas in our study exhibited a significantly higher ADC standard deviation than grade III gliomas. This information is significant for the oncologic surgeon, as the spatial distribution of different tumor grades in the neoplasm of the individual patient influences the strategy for resection, especially if the resection of the entire tumor is impossible without risking unjust surgical morbimortality.

Besides the aforementioned first order histogram characteristics like mean, median and percentiles, histogram analysis provides additional second order characteristics–kurtosis, skewness and entropy–which describe more complex aspects of the value distribution [13]. Increasing body of evidence suggests the superior value of additionally using these second order histogram dimensions for better reflection of tumor heterogeneity and associated tumor-biology [14–24]. In our study, glioblastomas were associated with significant higher positive skewness of the ADC histogram compared to grade III gliomas, which means generally a shifting of the ADC histogram curve with its peak towards lower ADC values, resulting in an asymmetrical histogram profile. On the other hand, entropy of ADC value distribution, an otherwise very promising histogram parameter in terms of reflecting tumor heterogeneity, did not show significant differences of mean values between tumor grades. This could be related to the similarity and partially overlapping tumor evolution of these tumor entities and skewness may be a better histogram parameter in order to differentiate tumor tissues in these cases. But this hypothesis needs to be evaluated in further studies. SD, another histogram parameter describing aspects of value distribution and especially value spread, exhibited different mean values between anaplastic astrocytomas and glioblastomas with strong statistical significance. Glioblastomas showed conspicuous higher SD values, a fact which is in line with the aforementioned differences in maximum and minimum values, describing a higher spread of the ADC continuum. The importance of SD of the ADC as a parameter reflecting tumor heterogeneity needs further corroboration, it is therefore commendable to include it into future histogram studies.

In line with the general accepted assumption, that higher glioma grade is associated with higher Ki-67 expression-based proliferation index, our study confirmed higher indices in grade IV glioma compared to grade III entities.

An important molecular property in gliomas bearing great prognostic relevance is the Oxygen 6-methylguanine DNA methyltransferase (MGMT) promotor methylation status. As crucial DNA repair enzyme it is capable of reversing naturally occurring DNA alterations and plays therefore an important role for genomic stability. MGMT gene silencing due to promotor methylation makes the affected tumor cells more sensitive to alkylating chemotherapeutics and leads to an increased overall survival in case of GBM [30]. In recent years, several imaging studies have been dedicated to ADC histogram profiling for a possible prediction of the MGMT promotor methylation status in GBM, but with conflicting results [31–35]. In line with these preexisting inconsistent data our actual study did not detect any significant difference in ADC histogram parameters regarding the MGMT methylation status.

Finally, correlation analysis revealed significant positive correlations between Ki-67 expression and ADC SD, ADCmax and Entropy as well as negative correlations between Ki-67 index and ADCmin and Kurtosis. These results are well consistent with the results of the comparative statistics, in which both the extreme values (ADCmin, ADCmax) and the SD differed significantly between the two tumor grades. Tumors with a higher tumor grade and thus a higher Ki-67 index appear to have a broader spread of ADC values. As far as entropy is concerned, a positive correlation between this parameter and the tumor proliferation rate could also be demonstrated in case of meningiomas [20]. The role of Kurtosis, which only exhibited borderline significance in the actual correlative statistics and whether it has the potential of predicting the proliferative activity of HGG has to be evaluated in further histogram analyses.

Our study has several limitations. Firstly, it was conducted as a retrospective study, which inevitably leads to an inferior level of evidence compared to prospective studies. Moreover, imaging data were obtained by only 1.5-T MRI devices and therefore exhibited a lower signal-to-noise ratio and consequently lower spatial resolution, which could have reduced the significance of the ADC histogram profiling in this investigation. Furthermore, ADC calculation was realized by employing only 2 b values (0 and 1000 s/mm^2^), which could have led to an increased impact of small vessel perfusion on ADC values in this study. Another potential limitation of our preliminary study is that the familywise alpha inflation due to serial hypothesis tests in the statistical analysis was not controlled. Finally, the neurosurgical specimen collection for further histological processing and diagnostics were almost exclusively performed by en-bloc resection. Related to the nature of the histopathological workup, a holistic analysis allowing a direct anatomical correlation with the histogram analysis, for example with neuronavigational data, was therefore impossible. However, the present study intended to use features of the whole tumor ADC volumes of presurgical MRI scans for correlation of prognostically relevant tumor biological features, but not a MRI–histopathology–volume correlation.

## Conclusion

ADC histogram profiling of HGG could facilitate in vivo differentiation of grade III from grade IV gliomas and furthermore enables to draw conclusions about the proliferative activity of the lesion at hand. As a consequence, ADC histogram profiling should be included in the initial presurgical diagnostics in case of suspected HGG. It could contribute to increase the accuracy of diagnosis and prognosis as well as help to detect possible hot spots of increased proliferation within the tumor tissue for targeted biopsy.

## Supporting information

S1 Table Primary histogram profiling parameters. S1 Table displays all histogram profiling parameters obtained on each patient level as well as the corresponding histopathological information.
(PDF)
