# Supplementary material for: Diffusion weighted imaging in high-grade gliomas: A histogram-based analysis of apparent diffusion coefficient profile
Source: PLoS One. 2021 Apr 15;16(4):e0249878. doi: 10.1371/journal.pone.0249878 (PMC8049265; doi:10.1371/journal.pone.0249878)
Supplement: S1 Table — displays all histogram profiling parameters obtained on each patient level as well as the corresponding histopathological information. (PDF) [file pone.0249878.s001.pdf]

Age (y)	Sex	Diagnosis	Mean ADC	Min ADC	Max ADC	P10 ADC	P25 ADC	P75 ADC	P90 ADC	Median ADC	Mode ADC	Std ADC	Kurtosis	Skewness	Entropy	IDH-1 mutation	MIB-1 (%)	MGMT methylation
48	m	Anapl. Astrocytoma III°	0.00146236	0.000656	0.002465	0.00123	0.001373	0.00157	0.001636	0.00149	0.001527	0.0001818	5.64233478	-0.4317704	5.07663113	1	10	1
61	m	Anapl. Astrocytoma III°	0.00142787	0.000528	0.003267	0.001038	0.001215	0.00162	0.001739	0.00145	0.001509	0.00031399	5.48662352	0.58427937	5.31622906	1	8	1
19	m	Anapl. Astrocytoma III°	0.00119148	0.000666	0.002229	0.0008851	0.000977	0.0013645	0.00163	0.001115	0.00107	0.00028288	2.88766919	0.81139208	4.34602241	x	x	x
41	m	Anapl. Astrocytoma III°	0.00147082	0.000675	0.002595	0.00111	0.001286	0.001663	0.00181	0.001478	0.001553	0.00027394	2.92651992	-0.0520741	4.48894617	0	12	x
30	m	Anapl. Astrocytoma III°	0.00125841	0.000578	0.002207	0.000937	0.001004	0.001545	0.00174	0.00113	0.001014	0.00031385	2.07546588	0.61666563	5.04565126	1	50	1
65	m	Anapl. Astrocytoma III°	0.00150186	0.000758	0.001978	0.001169	0.00132375	0.00169225	0.001792	0.001542	0.001622	0.0002469	2.72418368	-0.5368412	4.24231869	0	4	0
52	m	Anapl. Astrocytoma III°	0.00151004	0.000217	0.00265	0.001265	0.001382	0.001657	0.001747	0.001503	0.001454	0.00021388	6.23429652	0.25238761	4.7711481	0	3	x
28	f	Anapl. Astrocytoma III°	0.00186503	0.00069	0.002193	0.001607	0.001799	0.001996	0.002052	0.001919	0.001994	0.00020197	6.64107397	-1.7395368	5.015462	1	10	0
81	f	Anapl. Astrocytoma III°	0.00066562	0.000375	0.001423	0.000445	0.000498	0.000792	0.000999	0.0005985	0.000463	0.00021612	3.35081178	1.04024148	4.55219349	0	15	1
81	m	Anapl. Astrocytoma III°	0.00150803	0.001013	0.001838	0.001296	0.0014	0.001615	0.00172	0.0015155	0.001575	0.00015626	2.61739722	-0.1964171	4.37938837	0	10	0
68	f	Anapl. Astrocytoma III°	0.00119894	0.000586	0.00266	0.000968	0.001064	0.001311	0.00147	0.001182	0.001231	0.00018942	3.03258768	0.45533263	5.24782646	0	30	0
43	m	GBM	0.00123333	0.000001	0.002877	0.000865	0.000997	0.00139	0.0016961	0.001147	0.001079	0.00036738	5.40827956	1.33386684	4.68307935	0	20	0
81	m	GBM	0.00126836	0.000004	0.003404	0.000853	0.001034	0.001473	0.0017235	0.0012345	0.001211	0.00036755	4.28554228	0.57636388	4.87331556	0	30	1
69	m	GBM	0.00114982	0.000016	0.003115	0.000772	0.0009	0.001308	0.001593	0.001082	0.000948	0.00037697	6.43648238	1.46956859	5.48065688	0	10	0
67	m	GBM	0.00111386	0.000041	0.002666	0.0007278	0.00082	0.001373	0.001594	0.001025	0.000791	0.00035627	3.45658326	0.83003169	4.65252483	0	50	0
61	m	GBM	0.001617	0.000004	0.003049	0.000943	0.001141	0.002233	0.002716	0.001373	0.001232	0.00066834	2.10264901	0.59756219	4.22350942	0	x	1
78	m	GBM	0.00131975	0.000405	0.002809	0.000869	0.001018	0.001633	0.0018085	0.0012855	0.00106	0.00036035	2.0739838	0.19087849	4.77695337	0	20	0
49	w	GBM	0.00094003	0.000454	0.002865	0.000693	0.000771	0.001071	0.001228	0.000897	0.000765	0.00023264	8.47225303	1.50938758	4.80978932	0	15	1
69	m	GBM	0.00120664	0.00062	0.002391	0.000813	0.0009265	0.001473	0.0016223	0.001181	0.00081	0.00031727	2.28042333	0.34577577	4.5311965	0	20	0
59	m	GBM	0.00130175	0.000051	0.003081	0.000979	0.001128	0.001427	0.001619	0.001272	0.001317	0.00029009	8.25280042	1.52779203	5.13240507	0	50	0
71	w	GBM	0.00125198	0.000414	0.003093	0.000822	0.000997	0.001489	0.001631	0.001252	0.001502	0.00032821	4.01277528	0.50541161	5.38252389	0	80	0
73	w	GBM	0.00137547	0.000003	0.002688	0.001014	0.001185	0.001549	0.001713	0.001377	0.001376	0.00028303	3.95244189	0.31637068	5.24964469	0	25	1
74	w	GBM	0.0014474	0.000587	0.002927	0.001034	0.001163	0.00167	0.001941	0.001389	0.001272	0.00036068	3.17684964	0.74691126	4.76568426	0	20	1
58	m	GBM	0.00147804	0.000697	0.003028	0.001168	0.001258	0.001565	0.0019889	0.001381	0.001302	0.00035049	5.49897482	1.59878801	4.45761561	0	20	1
78	w	GBM	0.0018512	0.000616	0.003583	0.001109	0.001471	0.002216	0.002584	0.001866	0.001968	0.00052797	2.40418784	0.00152209	5.39271355	0	25	0
60	w	GBM	0.00134682	0.000586	0.003224	0.000952	0.001158	0.00151	0.001652	0.001359	0.001404	0.00031229	8.60663888	1.27777245	4.49207576	0	x	0
74	m	GBM	0.00158422	0.000001	0.003034	0.001075	0.001288	0.001845	0.002234	0.001533	0.001364	0.00043331	2.95631346	0.37147744	5.10246821	0	15	0
68	m	GBM	0.00147014	0.000257	0.003205	0.000994	0.001122	0.001709	0.002172	0.00133	0.00114	0.00048445	3.83358465	1.15715384	5.01086176	0	x	0
56	m	GBM	0.00110331	0.000323	0.00306	0.000827	0.000906	0.00118525	0.001429	0.00102	0.000868	0.00033415	10.9997537	2.49215383	3.25364596	0	15	1
60	w	GBM	0.00182102	0.000955	0.002715	0.001411	0.001556	0.002098	0.002254	0.001799	0.001484	0.00032406	2.11536983	0.03513185	4.64813116	0	20	1
47	m	GBM	0.00089796	0.000005	0.003385	0.00071	0.000781	0.000973	0.001104	0.000869	0.00083	0.0001908	17.5834123	2.4023606	5.03188833	0	40	0
84	m	GBM	0.00158281	0.000878	0.002998	0.001193	0.001306	0.001655	0.0023954	0.001442	0.001376	0.00044352	4.30703632	1.47554464	3.62434306	0	20	1
57	w	GBM	0.00138708	0.000006	0.003071	0.000939	0.001086	0.001633	0.001885	0.001337	0.00131	0.00038224	3.64159065	0.74786648	4.86324817	0	40	1
68	m	GBM	0.00138801	0.000759	0.003124	0.001073	0.001171	0.001619	0.001771	0.001342	0.001171	0.00026905	2.91619509	0.40701685	5.04255916	x	15	1
60	m	GBM	0.00137743	0.000002	0.003088	0.00092	0.00106	0.001555	0.002063	0.00126	0.001108	0.00047024	4.41915571	1.22726968	4.98200827	0	x	0
52	m	GBM	0.00113207	0.000523	0.002174	0.000767	0.000877	0.001368	0.00157	0.001083	0.00089	0.00030884	2.24906103	0.37929289	4.55030658	0	20	0
66	w	GBM	0.0007201	0.000422	0.001073	0.000631	0.000666	0.000753	0.0008279	0.000706	0.000687	8.7749E-05	4.97742448	1.08045685	3.99544967	0	15	1
62	w	GBM	0.00127453	0.000585	0.003034	0.000901	0.000987	0.001392	0.0017952	0.001166	0.000992	0.00042417	6.07234963	1.77676563	4.61985068	0	20	0
46	m	GBM	0.00144458	0.000001	0.003506	0.000854	0.001004	0.001638	0.002642	0.001248	0.001101	0.00061	3.17004711	1.12848604	5.11093853	0	30	0
66	w	GBM	0.00103747	0.000564	0.003607	0.00079	0.000868	0.00110275	0.00127	0.000985	0.00103	0.0003119	23.3384846	3.79930252	4.00051966	0	x	x
87	m	GBM	0.00097915	0.000482	0.002551	0.000721	0.000788	0.001065	0.001356	0.000895	0.000782	0.00029843	7.95582399	2.02894456	4.14009578	0	20	1
64	m	GBM	0.00102176	0.00056	0.003535	0.00078	0.000834	0.00114	0.001379	0.000935	0.000836	0.00027868	10.6620724	2.09962053	4.84304716	0	20	0
78	w	GBM	0.00127065	0.000034	0.002837	0.0008287	0.000957	0.001547	0.001746	0.001236	0.00086	0.0003775	3.0986996	0.48001661	4.84506769	0	20	0
78	m	GBM	0.00163715	0.000246	0.003434	0.000956	0.001128	0.002267	0.00257	0.001427	0.001155	0.00061767	1.83526197	0.48154922	5.23732247	0	40	0
72	m	GBM	0.00136565	0.00022	0.0032	0.000994	0.001104	0.001562	0.001862	0.001279	0.001104	0.00035848	4.42717007	1.15793746	4.67680567	0	20	1
69	m	GBM	0.00122364	0.000092	0.002746	0.000767	0.000854	0.001527	0.00193	0.001083	0.000779	0.0004427	2.57821954	0.80700349	5.54721908	0	70	1
76	w	GBM	0.00168826	0.000636	0.002958	0.001109	0.00124675	0.002168	0.002601	0.001471	0.001156	0.00055383	2.08261887	0.67465794	4.07382621	0	30	1
42	m	GBM	0.00188578	0.000297	0.003978	0.001096	0.001335	0.002563	0.002817	0.001748	0.00277	0.00065773	1.81348398	0.29282803	5.44722553	0	80	0
23	w	GBM	0.00183209	0.000195	0.003497	0.000894	0.001121	0.002711	0.002837	0.001636	0.002753	0.00077459	1.46245532	0.16768732	5.39492401	0	20	1
76	w	GBM	0.00188254	0.000459	0.003383	0.00097	0.00118	0.002672	0.002789	0.0017575	0.002741	0.00073947	1.35425722	0.03807542	4.24438317	0	60	0
50	w	GBM	0.0008178	0.000588	0.001391	0.000694	0.000744	0.000877	0.000968	0.000803	0.000795	0.00010789	4.4620897	0.85900344	3.80141415	0	10	0
62	w	GBM	0.00111789	0.000461	0.003395	0.000776	0.000876	0.001288	0.001533	0.001055	0.000877	0.00033268	8.15450693	1.63435749	5.27424337	0	25	0
64	w	GBM	0.00209181	0.000766	0.00361	0.001156	0.001347	0.00274	0.002826	0.002292	0.002721	0.00069676	1.42565777	-0.2329073	4.10846152	0	30	1
78	m	GBM	0.00155088	0.000041	0.002074	0.001346	0.001469	0.001665	0.001764	0.001574	0.001641	0.00020566	13.4836625	-2.0667831	3.85053629	0	20	0
47	w	GBM	0.00130816	0.000049	0.002858	0.000837	0.000962	0.001552	0.001905	0.00124	0.001006	0.00042567	3.32091825	0.82112287	4.95601524	0	20	1
79	m	GBM	0.00218477	0.000459	0.003684	0.000957	0.001467	0.00269	0.002826	0.002558	0.002665	0.00072803	2.11407585	-0.7933388	5.0793383	0	15	0
